# Diagnosis of malignant pleural disease: Ultrasound as “a detective probe”

**DOI:** 10.1111/1759-7714.14735

**Published:** 2022-11-22

**Authors:** Gaetana Messina, Mary Bove, Giovanni Natale, Vincenzo Di Filippo, Giorgia Opromolla, Anna Rainone, Beatrice Leonardi, Mario Martone, Alfonso Fiorelli, Giovanni Vicidomini, Mario Santini, Andrea Ronchi, Eva Massimilla, Carminia Maria Della Corte, Mario Pirozzi, Marianna Caterino, Fortunato Ciardiello, Morena Fasano

**Affiliations:** ^1^ Thoracic Surgery Unit Università degli Studi della Campania "Luigi Vanvitelli" Naples Italy; ^2^ Pathology Unit, Department of Mental and Physical Health and Preventive Medicine University of Campania Luigi Vanvitelli Naples Italy; ^3^ Otorhinolaryngology Unit Università degli Studi della Campania "Luigi Vanvitelli" Naples Italy; ^4^ Oncology, Department of Precision Medicine Università della Campania "L. Vanvitelli" Naples Italy

**Keywords:** malignant pleural mesothelioma, pleural biopsies, probe, thoracic ultrasound

## Abstract

**Background:**

Malignant pleural mesothelioma (MPM) is an invasive, aggressive pleural tumor with a predominantly local spread. The objective of this study was to assess thoracic ultrasound (TUS) as an imaging modality with high sensitivity for the identification of malignant pleural involvement and in order to guide pleural biopsies.

**Methods:**

In this retrospective single‐center study between January 2018 and June 2022, 51 consecutive patients with impassable circumferential pleural thickening underwent TUS at the Thoracic Surgery Unit of the Vanvitelli University of Naples. Pleural biopsies were performed, and then large and multiple samples were sent to the pathological anatomy for histological examination.

**Results:**

In all patients who underwent ultrasound examination, we chose the optimal point of entry to perform pleural biopsies and selected the areas of greater thickening without pleural effusion. No patient had any complications. No drainage tubes were placed after the pleural biopsies and no pneumothorax was present during the following days of hospitalization. The patients were discharged on the second postoperative day.

**Conclusion:**

With TUS the precise pleural thickening localization, local infiltration, mass extent, its nature (solid, cystic or complex) and ultrasound features can be easily defined. Furthermore, ultrasound is more economical than computed tomography and avoids the risks associated with radiation. Thoracic ultrasound is an important component of the diagnostic procedure in detecting a safe entry site for biopsies of MPMs.

## Graphical abstract

Chest ultrasound represents an important supplementary technique in the preoperative evaluation of the expansion of malignant pleural diseases. It allows to localize pleural tumors or pleural thickening and guide safe entry site for biopsy of the pleura, to assess the invasion of tumors to the pleura and chest wall, and guide biopsy of the tumors.

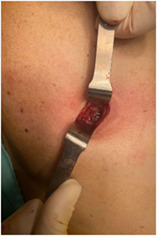

## INTRODUCTION

Malignant pleural mesothelioma (MPM) is an invasive, aggressive pleural tumor with a predominantly local spread that arises from the mesothelial cells^1^; it grows planarly between the parietal and visceral pleura and spreads by infiltration into the adjacent organs such as the diaphragm, chest wall, and mediastinum, but in some cases lymph node involvement and distant metastases may occur^2^; it is connected to professional asbestos exposure.^3^ There are four main histological subtypes: sarcomatoid, epithelioid, desmoplastic and biphasic or mixed.^4^ The epithelioid variant has a favorable prognosis with a median survival of 13.1 months,^5^, ^6^ while the sarcomatoid variant is associated with worse outcomes, with a median survival of only 4 months.^7^ Approximately 70% of MPM patients show pleural effusion^8^; however, diagnosis of MPM is difficult and cytology alone should not be enough for definitive diagnosis,^9^, ^10^ considering its very low sensitivity.^11^ Therefore, the diagnosis of MPM requires pleural biopsies and confirmation by expert pathologists according to European guidelines.^12^ In cases of suspected MPM, pleural biopsy is recommended to take large samples from the area of interest.^13^, ^14^ Although various guidelines prefer surgical thoracoscopic biopsy, this procedure is invasive and must be performed under general anesthesia with monopulmonary ventilation.^15^, ^16^ However, in many cases, pathological diagnosis remains difficult to obtain because certain patients are too frail to undergo thoracoscopy, and in some patients there is a circumferential pleural thickening with impassable pleural cavity. Therefore, the objective of this study was to assess thoracic ultrasound (TUS) as an imaging modality with high sensitivity for identification of malignant pleural involvement and to guide pleural biopsies to differentiate pleural primary from secondary malignancies.

## METHODS

The study was performed in compliance with the principles of the Declaration of Helsinki. Written informed consent was obtained from all participants during preoperative communication and the protocol was approved by the Ethics Committee of the University of “Luigi Vanvitelli” of Naples (n.280) on May 16, 2020.

All patients were hospitalized in our division, and the areas where to perform the TUS chosen according to the preliminary computed tomography (CT) scan. Inclusion criteria: patient with circumferential pleural thickening with inaccessible pleural cavity, respiratory and cardiac tests were within normal ranges, and no other preoperative comorbidities contraindicated an operation. Exclusion criteria patients with history of asthma, pulmonary fibrosis, chronic obstructive pulmonary disease, or severe heart disease, the presence of subcutaneous emphysema and obese patient with large thoracic dressings alters and precludes the propagation of the US.

**FIGURE 1:**
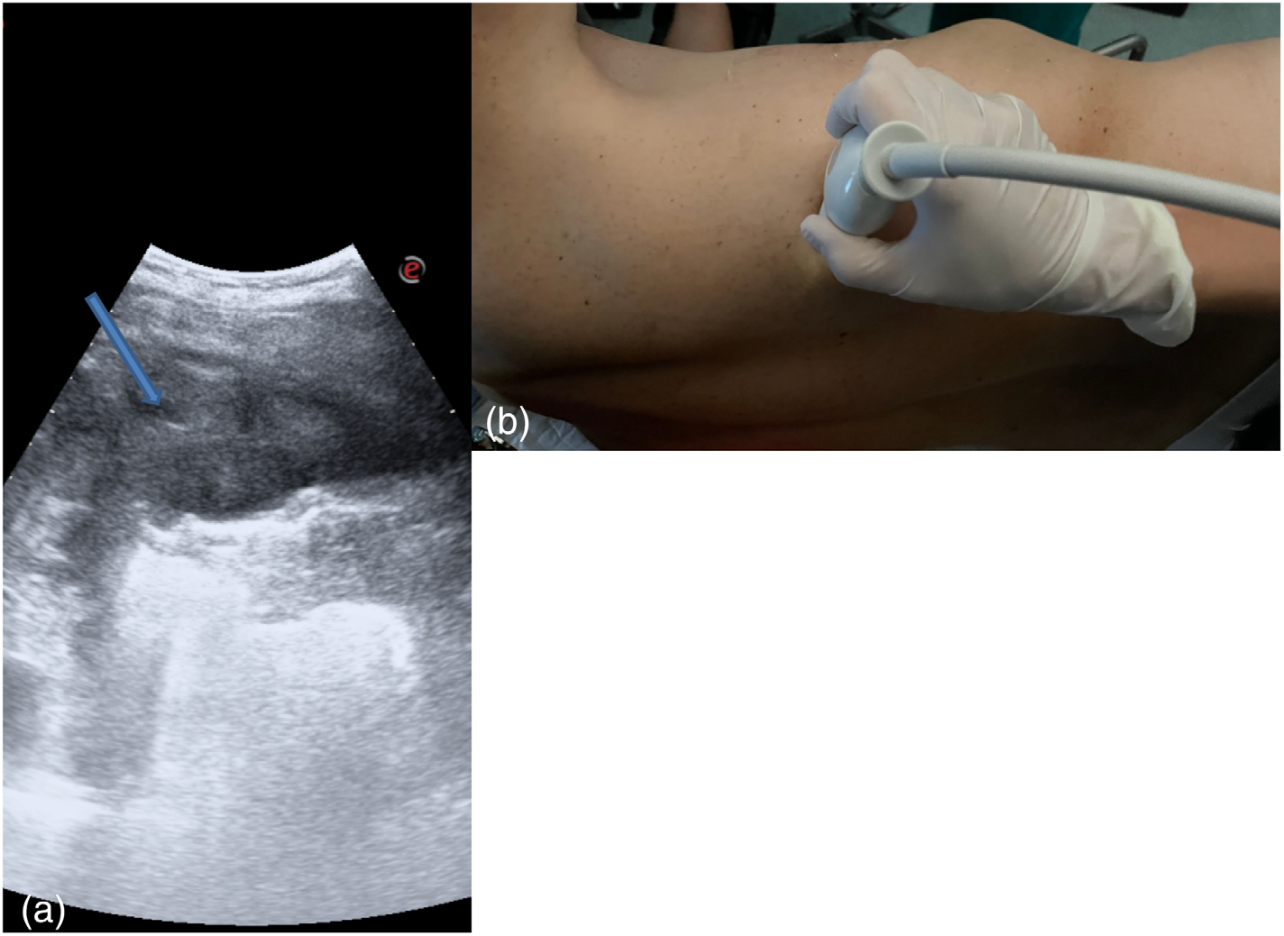
(a) Pleural thickenings were located with transverse or longitudinal scans, moving the probe along or across the intercostal spaces, avoiding interference by bony ribs. (b) Ultrasound information was obtained by tilting and moving the transducer along the intercostal spaces. Within each space two types of probe: convex and linear probes were used.

**FIGURE 2:**
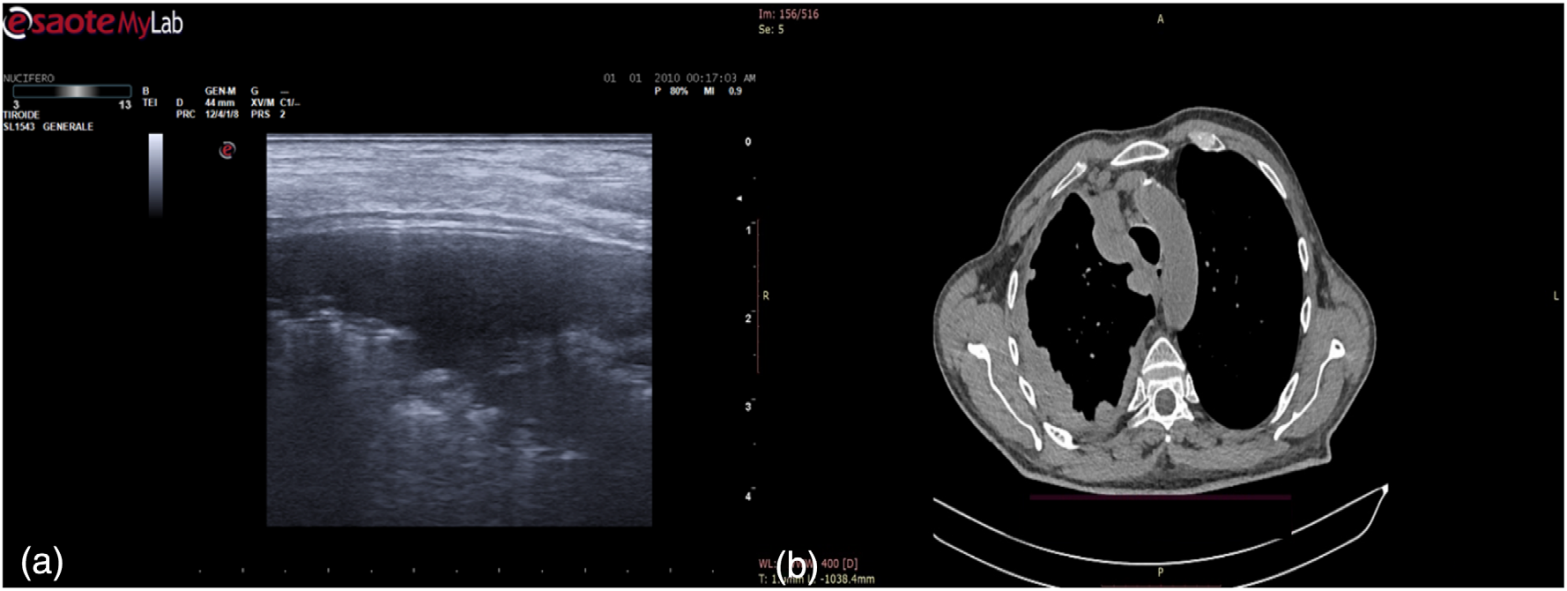
(a) Pleural thickening visualized as a lobular or oval hypoecogenic inhomogeneous structure, irregular shadow, hyperechoic spots and peripheral areas of these lesions may be characterized by an echo‐poor halo, central necrosis and spiculations. (b) Thoracic computed tomography (CT) shows circumferential pleural thickening.

**FIGURE 3:**
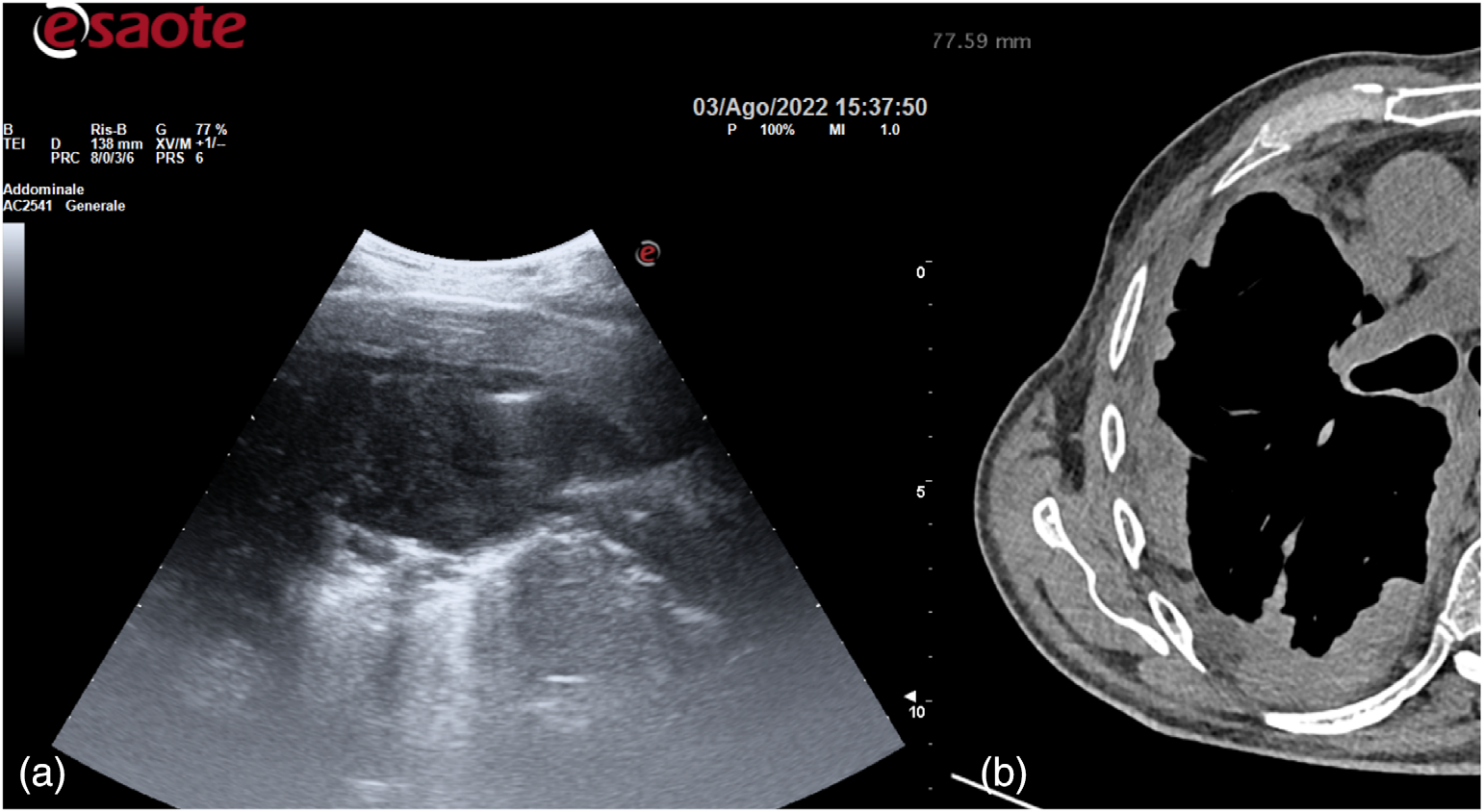
(a) An ultrasound study can easily define the precise pleural thickening localization, local infiltration, mass extent, its nature (solid, cystic or complex) and ultrasound features. (b) Thoracic computed tomography (CT) pleural circumferential thickening.

**FIGURE 4:**
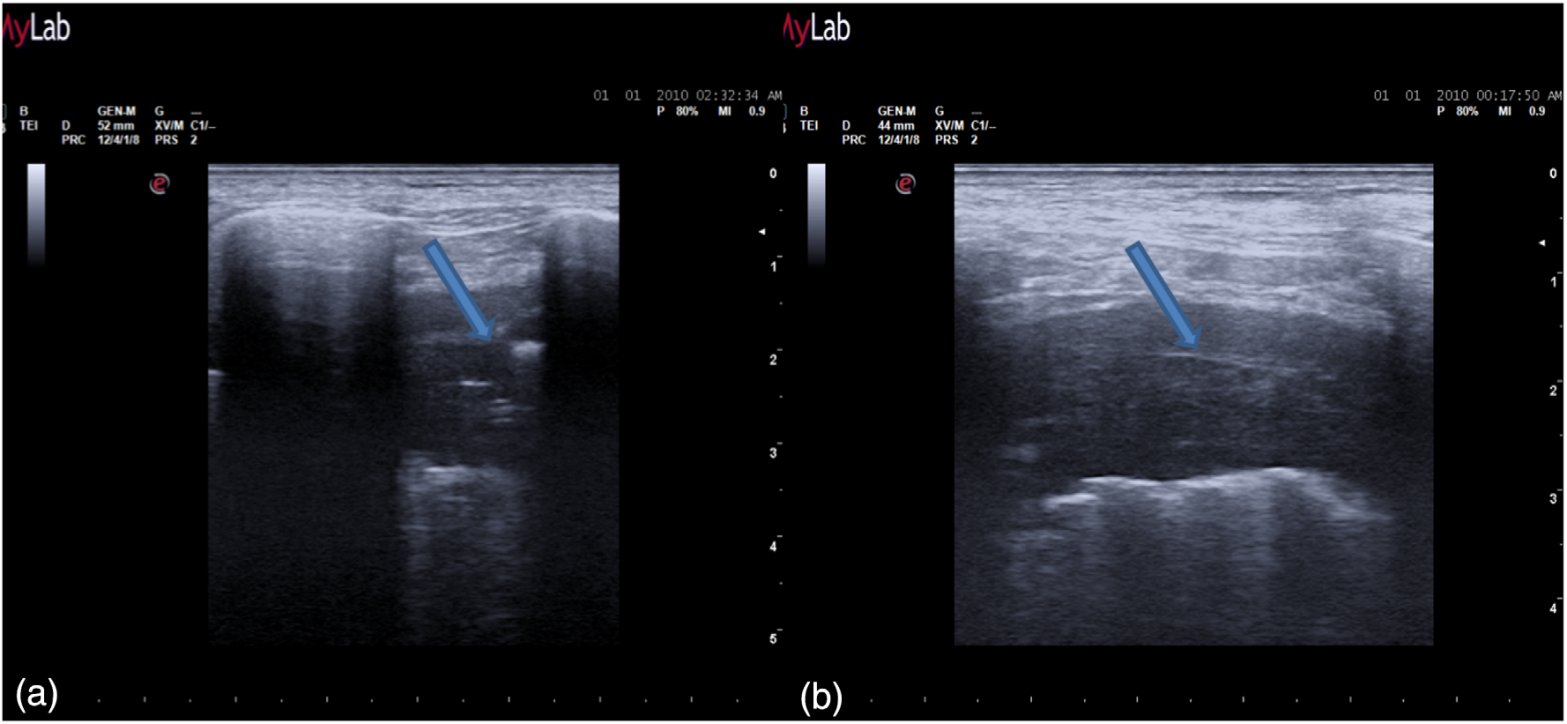
(a) In a longitudinal view the bat sign identifies the upper and lower ribs and, a little deeper, the pleural thickening. (b) An oblique approach allows visualization of a larger part of the pleural thickening, not interrupted by the rib shadows.

The day before the surgery, we systematically evaluated with ultrasound (US) the dorsal, lateral, and anterior chest wall to detect pleural thickenings, pleural nodules, and masses (Figure 1a). During chest US examination, we scanned patients in a sitting or supine position, while bedridden patients were examined by turning them to oblique or decubitus positions. We studied pleural thickenings with transverse or longitudinal scans, moving the probe along or across the intercostal spaces, avoiding interference by bony ribs (Figure 1b). Ultrasound information was obtained by tilting and moving along the intercostal spaces the transducer within each space. We used two types of probes: convex and linear (Figure 2). A convex probe has a low frequency (3–7 MHz), which creates a trapezoidal image more appropriate for visualization of deeper lesions with low resolution (Figure 3). A linear probe, with a high frequency transducer (7–15 MHz), creates a rectangular image which provides better visualization of surface lesions. The hemithorax was scanned circumferentially with US by laying the probe in the intercostal spaces, avoiding the ribs, in order to find a safe site of deeper pleural thickenings in which to perform a CT‐guided biopsy. Ultrasound scanning of the upper anterior, lower anterior and lateral chest was obtained scanning from the parasternal to the axillary line,^17^ while the posterior chest was scanned along the paravertebral, scapularis and posterior axillary lines. For scanning the anterior chest, the supine position is ideal whereas the lateral chest may be scanned in the semi‐supine position and for scanning the posterior chest the perfect position is with the patient sitting on the bed or prone. Lung ultrasound can be performed on the whole chest, just laying the probe in the intercostal spaces, avoiding the ribs. The probe can be positioned both longitudinally, perpendicular to the ribs, and obliquely, along the intercostal spaces. In the longitudinal view the bat sign identifies the upper and lower ribs and, a little deeper, the pleural thickening (Figure 4a). The oblique approach allows visualization of a larger part of the pleural thickening, which is not interrupted by rib shadows (Figure 4b). Pleural thickening may be visualized as lobular or oval hypogenic inhomogeneous structure, irregular hyperechoic spots and peripheral areas of these lesions may be characterized by an echo‐poor, central necrosis and spiculations.

In addition, chest ultrasonography was performed on the day of the procedure when the patient was typically positioned in the lateral decubitus position with the affected side upward under sedation and spontaneous breathing, with an ESP block.

**FIGURE 5:**
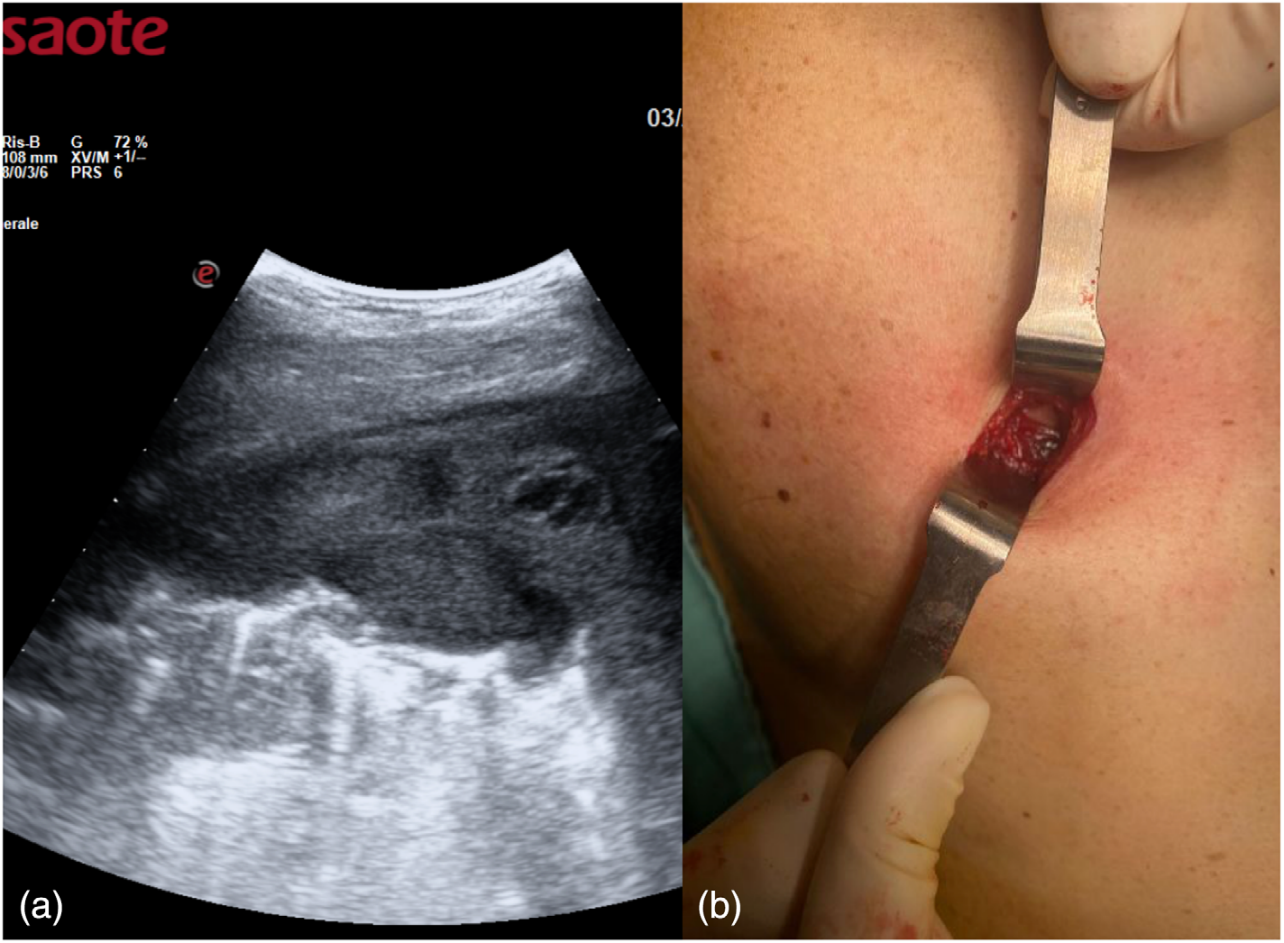
(a) We searched for areas with greater pleural thickening and a better entry site. (b) Once the target was defined in terms of size, shape, echogenicity and vascularization, the main operator faced the patient, allowing an optimal approach to the parietal pleura. Local anesthesia was induced with mepivacaine and followed by a small skin of about 2 cm incision, the pleural biopsies were slowly performed.

**FIGURE 6:**
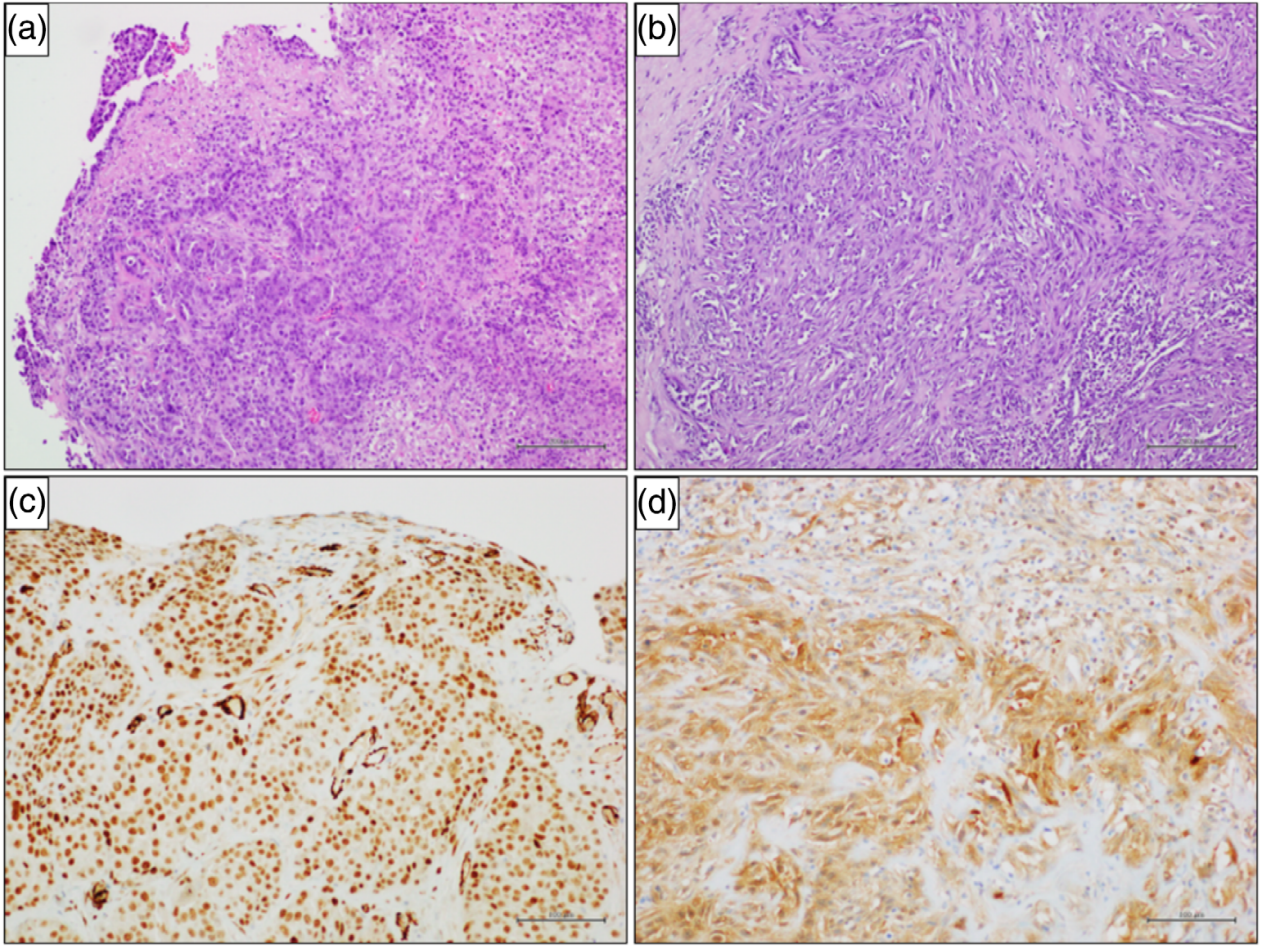
Pleural biopsies showing a neoplastic population diffusely infiltrating the fibrous stroma. (a) The neoplastic population constituted epithelioid (hematoxylin and eosin stain, original magnification 100×) or (b) sarcomatoid (hematoxylin and eosin stain, original magnification 100×) cells. Immunohistochemistry showed (c) positivity for WT1 (immunohistochemical stain, original magnification 200×) and (d) calretinin (immunohistochemical stain, original magnification 200×).

A US study previously easily defined the precise pleural thickening localization, local infiltration, mass extent, its nature (solid, cystic or complex) and US features.^18^ Thoracic US can also guide biopsy of pleural tumors if an appropriate and safe window is identified.^19^ We searched for areas with greater pleural hyperextension and strongly suspected to fix the better entry site. Once the target was defined in terms of size, shape, echogenicity and vascularization, the main operator faced the patient, allowing an optimal approach to the parietal pleura, a small skin incision of about 2 cm was made, and an incisional biopsy was slowly performed using forceps (Figure 5). Multiple samples were then sent to pathological anatomy for histological examination (Figure 6).

### Statistical analysis

The true positive were 35, the false positive were 2, the false negative was 1. Therefore, in this series, the sensitivity, PPV, NPV and accuracy were 97, 95, 93 and 94%, respectively (Table 1).

**TABLE 1:** Statistical analysis

Method	Sensitivity (%)	Positive predictive value (%)	Negative predictive value (%)	Accuracy (%)
Ultrasound	97	95	93	94

## RESULTS

The primary aim of this observational retrospective single‐center study was to confirm the validity of TUS as a safe and effective method of localization of malignant pleural involvement.

From January 2018 to June 2022 at the Thoracic Surgery Department of the Vanvitelli University of Naples, 51 consecutive patients with impassable circumferential pleural thickening underwent TUS. They had chest radiographs taken a maximum of 3 days before pleural biopsies and a thoracic CT scan taken a maximum of 2 weeks before pleural biopsies. They subsequently received detailed information about the potential risks associated with the procedure.

Patients were eligible if a chest CT scan was performed within 2 weeks of the biopsy procedure, and chest radiographs taken a maximum of 3 days before pleural biopsies. The mean age of the patient cohort was 69 (±10.7 years); 38 patients were men, and 13 were women. The right side of the chest was more generally explored than the left side (62% vs. 38%, respectively). A total of 23 patients (45%) were smokers and 41 (80%) had a history of occupational asbestos exposure. Clinical manifestations are commonly nonspecific and insidious, with mild breathlessness, nonspecific thoracic pain, weight loss, fatigue. A total of 43 patients (85%) had a final diagnosis of MPM and seven (15%) had a final diagnosis of secondary tumor. MPM subtypes were epithelioid in 31 cases (71%), sarcomatous in seven cases (16%), mixed in four (10%), and desmoplastic in one (3%). Among the seven (15%) patients with secondary tumor, primary malignancies were lung cancer in four (70%) patients, breast cancer in one (10%), squamous cell carcinoma of unknown origin in one (10%), and inflammatory thickening in one (10%), respectively. With US examination, we chose the optimal entry point to perform pleural biopsies and chose areas of greater thickening without pleural effusion. In all patients, pleural mesothelioma presented ultrasonographically as an irregularly limited, echo‐poor, knotty or planar widening along the pleura; in this series, the sensitivity, PPV, NPV and accuracy were 97, 95, 93, and 94%, respectively. No patient had any complications such as bleeding, infection, damage to the pleura visceralis, pneumothorax, hemoptysis, furthermore, US is more economical than CT and avoids the risk associated with radiation. No drainage tubes were placed after the pleural biopsies and no pneumothorax was present during the following days of hospitalization. The patients were discharged on the second postoperative day (Table 2).

**TABLE 2:** Patient characteristics

Characteristics	n (%)
Patients, *n*	51
Age, years (mean)	69
Sex (male), *n* (%)	38 (75%)
Smokers	21 (45%)
Asbestos exposure	41 (80%)
Histology, *n* (%)	
Epithelioid mesothelioma	31 (71%)
Sarcomatoid mesothelioma	7 (16%)
Mixed mesothelioma	4 (10%)
Desmoplastic mesothelioma	1 (3%)
Lung cancer metastasis	4 (70%)
Breast cancer metastasis	1 (10%)
Squamous carcinoma of unknown origin	1 (10%)
Ovarian cancer	1 (10%)
Chest drain	0
Hemorrage	0
Pneumothorax	0
Days of hospitalization	2 ± 1

## DISCUSSION

Assessment of the lung has previously been considered off limits for US, «because ultrasound energy is quickly dissipated by air, therefore ultrasound imaging is not useful for the evaluation of the pulmonary parenchyma».^20^ Ultrasound cannot be used for evaluating the lung: it is connected to the presence of air, which determines a total acoustic mismatch with the surrounding tissues, causing a total reflection of the US.^21^

An increase in the pleural thickness, however, suggests the likelihood of malignancy.^22^ A widening of the pleura of more than 1 cm is considered strongly indicative of the presence of a malignant tumor.^23^ Pleural mesothelioma presents ultrasonographically as an irregularly limited, echo‐poor, knotty or planar widening along the pleura. Thus, early testing to discriminate between benign and malignant pleural disease is important for effective treatment, as well as extending survival. CT features such as pleural nodularity, pleural thickening more than 1 cm and circumferential pleural disease are highly sensitive for the diagnosis.^24^ Chest X‐ray usually reveals pleural thickening.^25^ Diffuse, irregular, or nodular pleural thickening, parietal, mediastinal, fissure, and/or diaphragmatic pleural involvement has been previously reported on chest CT scan.^26^ Chest magnetic resonance imaging has not previously been deemed a relevant diagnostic tool.^27^ Positron emission tomography/CT scanning has previously been reported to be helpful in determining the local extent of pleural thickening.^28^, ^29^ Chest US is very useful in identifying pleural thickenings or nodules.^30^ A large retrospective review performed in patients with mediastinal pleural thickening, nodular pleural thickening, parietal pleural thickening greater than 1 cm and circumferential pleural thickening, reported a 68% sensitivity and 78% specificity in predicting malignancy.^31^ Focused US of the pleura can be used to demonstrate pleural, nodularity pleural thickening and malignant invasion. CT has been shown to have poor sensitivity in detecting malignant chest wall invasion while TUS is superior^32^ and can predict malignant involvement of the pleura and chest wall.^33^ In a previous study, US color doppler has been used to differentiate between small pleural effusions and pleural thickening with 89% sensitivity and 100% specificity.^34^ Metintas et al.^35^ studied the prevalence of circumferential pleural thickening in MPM and compared it to that in secondary pleural malignancy. Their results were consistent with 70% of circumferential pleural thickening in MPM. In another study which included benign pathologies, Armato et al.^36^ obtained a prevalence of 73% for circumferential pleural thickening in their MPM group versus 33% in their secondary pleural malignancy group. Despite the small number of patients, chest US has some advantages, such as US‐guided pleural biopsy in cases when the pleural involvement may be focal. Because focal pleural thickening or pleural tumors can be easily identified with US, the pleural biopsy can aim at the focal area with ultrasonographic abnormalities.

Medical thoracoscopy is indicated for the evaluation of pleural effusions of unknown etiology and of pleural thickening, performing histological diagnosis and staging of pleural malignancies.^37^, ^38^ When the pleural thickening is circumferential and makes pleural cavity impassable, medical thoracoscopy is not possible. For this reason, we perform a mini thoracotomy and extrapleural biopsies.

Compared to tru‐cut, this method allows more significant biopsy samples to be obtained. It also allows exact measurement of the pleural thickening and the site that is ultrasonographically suspicious for malignancy in real time in the operating room to be identified, while without US the surgeon chooses the site of the biopsy only on the anatomical landmarks of CT images. The CT guided mark of most suspicious site is possible, but the decubitus of the patient on the operating table can modify the site previously marked under CT guide.^39^ Ultrasound also reduces complications such as pneumothorax and hemorrhages.

The pleural thickenings can be carefully visualized, located, and marked with US before the procedure is done. The lesion can even be monitored with dynamic images of real‐time US during these procedures. The main contraindication of these procedures is hemorrhagic diathesis. Ultrasound is more economical than CT and avoids the risk associated with radiation. Although the risk of complications decreases with US guidance, these procedures should still be performed with caution in patients with borderline respiratory failure. The chances of obtaining pleural tissues with significant pathological findings will thus increase. This is particularly true for patients even without pleural effusion.^40^, ^41^

Ultrasound scanning is both operator and patient dependent. It is only possible to obtain images of areas where the probe can be placed and the US waves can penetrate. An inexperienced operator may not obtain or record adequate images and can be misled by commonly produced artifacts in the scan. Chest US has some limitations, such as subcutaneous emphysema or pleural calcifications which may block US wave propagation, thus preventing the lungs from being seen, and due to the intrinsic characteristics of the chest wall (presence of bones), only 70% of the pleural surface can be explored. Delivering training in US to meet these standards has been challenging, but it is now part of the thoracic surgery training curriculum.

In conclusion, in our opinion, chest US represents an important supplementary technique in the preoperative evaluation of the expansion of malignant pleural diseases. It allows pleural tumors or pleural thickening to be localized and guides safe entry site for biopsy of the pleura, to assess the invasion of tumors to the pleura and chest wall, and guide biopsy of the tumors. Also, the US helps to clarify the cause of pleural opacities, to differentiate between minimal pleural effusion and pleural thickening. Additional advantages of US include the absence of radiation, low cost, flexibility and, in addition to availability and short examination time, provides immediate, real‐time diagnostic information allowing image‐guided chest interventions, with minimal to no risk to the patient. US is notably helpful for critically ill patients because of its portability and simplicity. In future, training in thoracic US may become as important as use of the stethoscope for respiratory and pleural practice and US will become like a “probe detective” for diagnosis of malignant pleural disease.

In conclusion, this was a single center preliminary study that needs further cases to corroborate our data.

## AUTHOR CONTRIBUTIONS

Concept and design: Gaetana Messina, Mary Bove, Anna Rainone. Administrative support: Fortunato Ciardiello, Morena Fasano. Provision of study materials or patients: Alfonso Fiorelli, Giovanni Vicidomini, Mario Santini. Collection and assembly of data: Mario Martone, Mario Pirozzi, Carminia Maria Della Corte, Marianna Caterino, Andrea Ronchi, Beatrice Leonardi. Data analysis and interpretation: Giovanni Natale, Giorgia Opromolla, Vincenzo Di Filippo, Eva Massimilla. Manuscript writing: all authors. Final approval of manuscript: all authors.

## CONFLICT OF INTEREST

All authors have completed the ICMJE uniform disclosure form. The authors have no conflicts of interest to declare.
